# Assessment of prevalence of *Giardia lamblia* infection and its associated factors among government elementary school children from Sidama zone, SNNPR, Ethiopia

**DOI:** 10.1371/journal.pone.0264812

**Published:** 2022-03-15

**Authors:** Sunil Tulshiram Hajare, Yeinewub Chekol, Nitin Mahendra Chauhan

**Affiliations:** Department of Biology, College of Natural and Computational Sciences, Dilla University, Dilla, Ethiopia; Abadan University of Medical Sciences, ISLAMIC REPUBLIC OF IRAN

## Abstract

Giardiasis is a protozoan disease caused by the parasite *Giardia lamblia*. Around 200 million people are infected worldwide annually while, 500,000 deaths were reported each year. The infection rates were between 2–5% in the developed nations and 20–30% in the developing countries. The parasite is associated with poverty, poor sanitation, lack of clean and safe drinking water supply, and poor personal hygiene. The aim of our study was to assess the prevalence rate and associated risk factors of *G*. *lamblia* infection among the elementary school children at Loka Abaya town, Ethiopia. A cross-sectional study was conducted from December 2018 to July 2019. A total number of 422 students were selected by using simple random sampling. Structured and semi-structured questionnaire was used to identify known risk factors such as environmental, socio-demographic, and behavioural. Stool specimens were collected from the study subjects and examined using direct smear method, microscopically. A bivariate and multivariate logistic regression analysis was done. *P* value less than 0.05 at 95% of the confidence interval was considered statistically significant. The overall prevalence rates of *G*. *lamblia* infection were 27.1%. Rural school from Aregeda [AOR: 9.997, *P* = 0.005], age group of 6–9 years [AOR: 2.305, *P* = 0.019], consuming tap water [AOR: 0.011, *P =* 0.006], hand washing habit after defecation with water only [AOR = 0.313, *P* = 0.040], use of soap and water [AOR: 0.046, *P* = 0.000] were the factors which are found to be associated with the parasite infection when compared to urban school. As such, the prevalence of *G*. *lamblia* infection was found to be high in the studied area among school children. Thus, scaling-up of access to safe water, toilets, education, and health facilities are required to possibly eradicate this predicament.

## Introduction

Giardiasis is a common gastrointestinal disease, caused by the flagellate protozoan Parasite *Giardia lamblia*. *Gardia lamblia*, which is also recognized as *Giardia intestinalis* or *Giardia duodenalis*, is the most common protozoan infecting the small intestine of humans and is a major cause of enteric infection throughout the world, especially in children [1]. *G*. *lamblia* is ranked among the top 10 parasites of man [2]. Around 200 million people are infected worldwide annually while 500,000 deaths are reported per year [3, 4]. According to World Health Organization about 200 million people in Asia, Africa and Latin America showed symptoms of giardiasis with about 500,000 new cases every year especially in children. In Ethiopia 10–50% of giardiasis infection was reported mostly in school children [5]. Studies on prevalence rate of the *G*. *lamblia* infection at different tropical as well as sub tropical areas have shown that they are highly prevalent and have great impact on human health [5].

*Gardia lamblia* has two morphological forms, cyst, and trophozoite. Trophozoites are pear-shaped, bi nucleate, multi-flagellated parasite forms capable of division by asexual binary fission. This form is the active, motile feeding stage that caused the pathology in the small intestine. The cyst forms are non-motile, resistant, stable stages, which don’t adhere to the mucosal surface [6]. According to Kayser et al. [1], trophozoites are responsible for producing disease in humans by attaching themselves to the walls of the small intestine, followed by rapid multiplication. Parasite cyst forms are not ably infectious in nature and are mainly responsible for the transmission of disease. The cyst stage is resistant to different concentrations of chlorine used in water treatment plants. Infection caused by cyst stage exists in 50% of the symptomatic carriage and reserves the infection in endemic form [7].

Definitive diagnosis of *G*. *lamblia* is finding the trophozoite or the cyst forms during microscopic examination of stool. Macroscopically, the stool is usually offensive, bulky, pale, non-bloody, mucoid (fatty) or watery [8]. The symptoms of Giardiasis in humans are extremely variable. Some people may present asymptomatic form, while, others may produce acute or chronic diarrhea that can last for several months with malabsorbtion, cholecystitis, and weight loss [9].

The major causes of infection are the consumption of inadequately treated contaminated water, ingestion of contaminated undercooked vegetables or unwashed fruits, and person-to-person spread by the faecal-oral route. Prevalence rate is high in areas with poor sanitation and varies from 2% to 5% in developed to 20% to 30% in developing countries. The variation in prevalence might be attributed to factors such as the geographical area, the urban or rural setting of the society, the age group composition, and the socio economic conditions of the study subjects [10]. Transmission of *G*. *lamblia* occurs mainly through contaminated water and food. Other factors includes: poor living conditions, overcrowded housing, poor environmental sanitation, unhygienic personal habits, unsafe water supply, and low socioeconomic conditions [1].

Most of the *G*. *lamblia* infections are more severe in children when compared to adults, which is associated with malnutrition, growth retardation, and poor care [11]. School children carry the heaviest burden of the associated morbidity due to their poor personal hygiene, unhygienic toilet practices, consuming unwashed fruits and undercooked vegetables, drinking, and eating of contaminated water and food [12]. In Ethiopia *G*. *lamblia* infection is one of the most common parasitic infections in the schools. For example, the prevalence rate at Dona Berber primary school in Bahir Dar was found to be 11.4% [13], in Homesha District it was observed as 12.65% [14], Wukro town from Eastern Tigray reported 16.9% [15], Lake Zuway elementary school has recorded 4.69% [16], Southeast of Lake Langano estimated 6.2% [17], in MizanTepi elementary schools it was noted 5% [18], and in Arba Minch it was calculated as 4.2% [12].

Even though several studies have been conducted on the distribution and prevalence of Giardiasis in Ethiopia, there are still several localities for which epidemiological information is not available including Sidama zone, SNNPR, Ethiopia. Therefore, this work is the first approach to determine the prevalence of *G*. *lamblia* infection and its associated factors from selected area. Results of this work are discussed below.

## Materials and methods

### Study area

The study was conducted in Loka Abaya town, Sidama zone, Ethiopia. It is located in the Southern Nation Nationalities Peoples Region (SNNPR) of Ethiopia and a part of Sidama zone located in the great rift valley of Ethiopia and has a tourist attraction namely as Loka Abaya National Park. The place is located at a distance of 344 km towards South East of the capital city of Ethiopia i.e. Addis Ababa and 69 km from the regional capital i.e. Hawassa. The place lies within the range between 6.42°-6.83° latitude and 38.01°-38.36° longitude with an elevation of the range between 1182–1867 meters above sea level. A total number of 27 elementary schools are there in the proposed areas. Most of the students in each school have an age above 6 years and below 18 years [19].

The toilet coverage in the study area is very less and the waste disposal system is very poor. As such, they dispose of the waste most often in the open field. Access to clean water was also found to be limited in this area. As a result of this, people are forced to use various unprotected water sources such as river, stream, rain, and pond water. The handling practice of water was poor as they use it directly because of the lack of awareness about the transmission of intestinal parasitic. But *G*. *lamblia* infection is the most common parasitic disease reported in the study area [19].

### Study design

A school based cross-sectional study was conducted from December 2018 to July 2019 to determine the prevalence rate of *G*. *lamblia* infection and associated risk factors among students from three selected government elementary schools namely, Hantate, Chelbesa and Aregeda situated in Loka Abaya town, Sidama zone, SNNPR, Ethiopia.

### Source of population

The source of population was all primary school students found in Loka Abaya town, Sidama zone, SNNPR, Ethiopia. The study population were school children admitted in grade 1 to grade 8 enrolled in three selected elementary schools cited above. The total numbers of enrolled students in 2018/19 academic year were 4804 (2374 males and 2430 females).

### Sample size determination

According to Daniel [20], a single population proportion formula was used to calculate the sample size by considering the following statistical assumptions:

$$\text{n}={\text{Z}}^{2}\;\text{P}\;\left(1-\text{P}\right)/{\text{d}}^{2}$$

Where: n = sample size, Z = the corresponding Z score of 95% Confidence Interval (1.96); D = Margin of error (5%); P = expected prevalence rate of *G*. *lamblia* infections in the study area. Since no report was recorded for prevalence of *G*. *lamblia* infection in the study area and to maximize the sample size, P = 50%. Accordingly, **N = (1.96)**^**2**^ **x 0.5 (1–0.5)/(0.05)**^**2**^ **= 384**. To compensate for the non-respondents and to minimize errors probably arising from the likelihood of non-compliance 10% was added giving a final sample size of 422 [21].

### Sampling technique and procedures

Elementary schools in Loka Abaya town were selected randomly. Then, three government elementary schools namely Hantate, Chelbesa and Aregeda among twenty-seven schools were identified by simple random sampling method. The number of students in Hantate, Chelbesa and Aregeda elementary schools were 2084, 1594 and 1126, respectively. After identifying the number of students in each school, the sample size was allocated proportionally to select the study participants from each school studying in grade 1 to grade 8. Based on their grade level, students were classified into eight strata. From each stratum, students were selected by proportionate allocation technique [22]. All preschool children who started taking anti-parasitic drugs and those who completed treatment before three days were excluded from the study.

**Fig 1:**
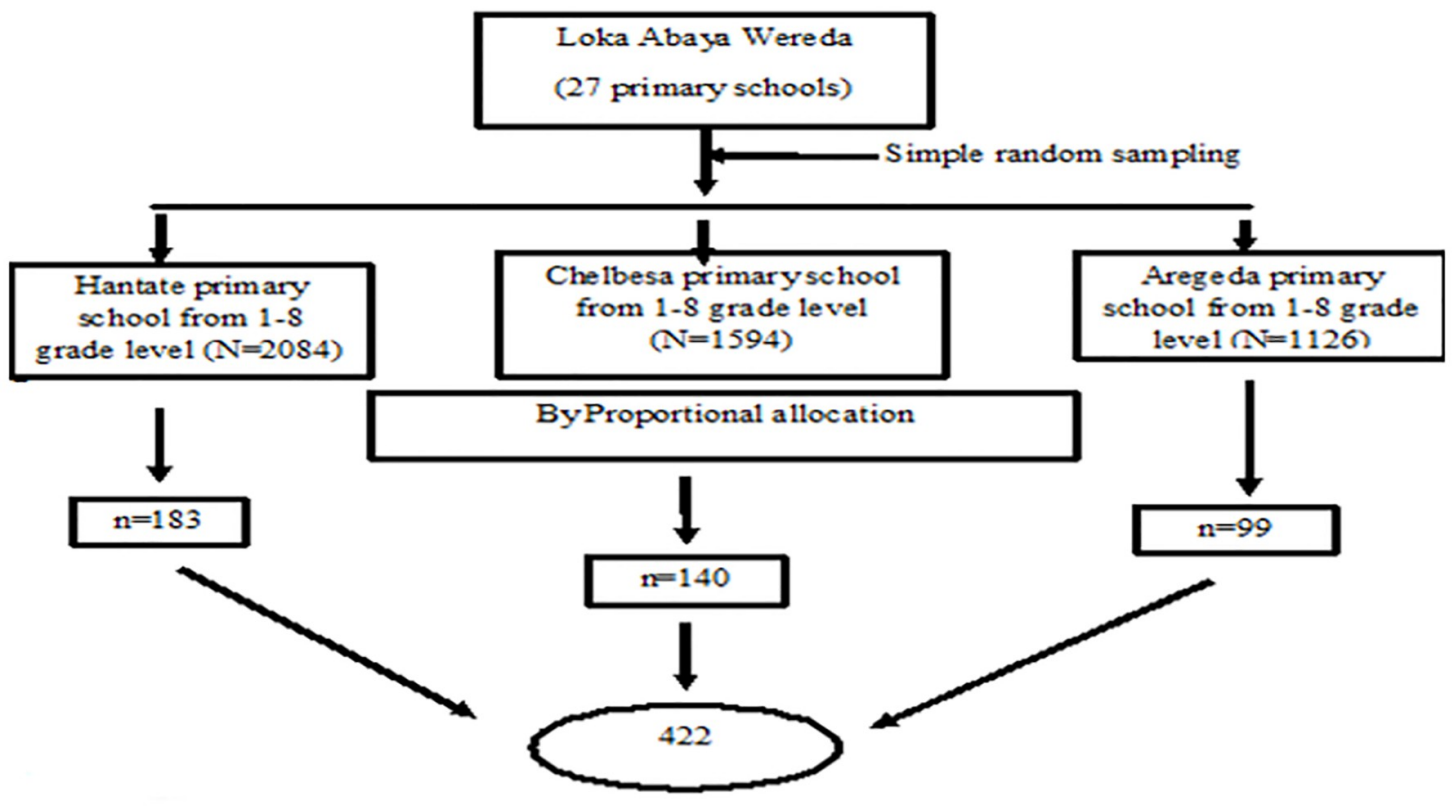
Schematic presentation of sampling procedure to determine the prevalence and associated risk factors of *G*. *lamblia* infections from selected government elementary schools located in Loka Abaya town, Sidama zone, SNNPR, Ethiopia.

According to Kothari [23], the selection of participants in every class was done with systematic sampling method using class roster as follows. First, numbering the grade levels of each school on the sample frame from 1 to N (N = Total number of students in each grade levels), then the sampling interval (K) was determined by dividing the number of students in each grade levels by the desired sample size of each class (n = sample size of each class) which gives a size of eleven. Finally from the number between 1 and 11, number 5 was selected randomly. This number is called the random start and the first number included in the sample. Then later selection was conducted every 2^nd^ unit after that first number until the desired number was obtain (Table 1; Fig 1).

**Table 1:** Selection of number of participants in each school from grade level 1 to 8 by proportion allocation method.

Name of school	Total number of students in each school	Study level of grade	Total number of students in each grade	Total number of students selected from each grade
Hantate	2084	Grade 1	270	33
		Grade 2	256	23
		Grade 3	218	28
		Grade 4	272	24
		Grade 5	290	25
		Grade 6	283	16
		Grade 7	210	18
		Grade 8	285	16
Chelbesa	1594	Grade 1	219	28
		Grade 2	206	27
		Grade 3	248	22
		Grade 4	211	18
		Grade 5	272	15
		Grade 6	227	11
		Grade 7	111	10
		Grade 8	100	09
Aregeda	1126	Grade 1	192	17
		Grade 2	162	14
		Grade 3	150	13
		Grade 4	140	12
		Grade 5	117	10
		Grade 6	130	12
		Grade 7	119	11
		Grade 8	116	10

### Study variables

#### Dependent variable

*Giardia lamblia* infection (Positive or Negative) was used as a dependant variable in the proposed work.

#### Independent variable

Independent variables in the form of socio-demographic factors like age, sex, residency or school setting, family education, family occupation, house hold members; Environmental factors such as source of water, availability of toilet, sanitary condition, disposal off house hold waste; Behavioural factors include washing of hands before eating, washing of hands after defecation, cooking of vegetables and washing fruits before consumption, water storage, treatment of water, habit of nail trimming were considered in the study.

### Data collection tools

To assure the reliability of data collection for the study structured and semi-structured questionnaire were prepared originally in English language and then translated into the national Amharic language and local Sidamagna language. After data collection, the data collected in Sidamagna and Amharic languages were retranslated into English by taking the help of expertise to assure consistency. The researcher was requested to interview their students and fill the questionnaires. Stick, handbook, glove, and labelled stool cup were used for the data collection. Both close and semi open-ended questions were utilized to collect data related to socio-demographic, environmental, and behavioural risk factors associated with *G*. *lamblia* infection. At the time of collection, date of sampling, number of the participant, grade, section, school name, age and sex were recorded.

### Collection of stool samples and laboratory analysis

According to Cheesbrough [24], after proper instruction, each student was provided with clean, dry, and leak-proof stool cup with unique identification number along with pieces of applicator sticks to bring proper fresh stool specimen. All students were instructed to bring a lot of stool samples so that no contaminates can be mixed. On delivery of the stool specimen, each student was interviewed using structured and semi-structured questionnaire for socio-demographic and related risk factors for *G*. *lamblia* infection. After the stool samples were collected, all specimens were subjected to direct wet mounts and formol-ether concentration technique [25]. About 2 mg of stool sample was gently mixed with a drop of normal saline on a microscopic glass slide finally covered with a cover slip and examined using microscope (Olympus BH-2). After completion of preliminary stool examination, samples were preserved in a 10% formalin solution and immediately transported to Parasitology Laboratory, Dilla University, Dilla, Ethiopia, to perform microscopic examination [25]. Briefly, the preserved tool sample was sieved with cotton gauze and transferred to 15ml centrifuge tube. Then 7 ml of 10% formalin and 3 ml of ethyl/ether acetate was added and centrifuged for 2 minutes at 2000rpm. The supernatant was decanted and the residues were transferred to microscope slides and observed under a light microscope at 100 X and 400 X magnifications for the presence of cysts and ova of the parasites. All standard protocols were followed strictly for stool sample examination to confirm the quality and sensitivity of the test result.

### Ethical approval and consent to participate

The study was reviewed and approved by the ethical committee of Biology department, Dilla University, Dilla, Ethiopia. Ethical considerations were addressed by treating positive intestinal protozoa by giving the drug of choice freely under the prescription and clinical supervision by an authorized health professionals at study sites. The questionnaires concerning the prevalence of study were filled during sample collection. Written consent was obtained from the parents or guardians of the selected study children. Apart from these, parents or caregivers of children were asked to fill out the questionnaire and assist the children during sample collection. The information obtained during the course of study was kept confidential.

### Data analysis

Data was entered in to Epi data version 4.4.2.1 and exported into SPSS version 21 for analysis. The baseline characteristics of the study population were summarized using frequencies or percentage into categorical variables and continuous variables expressed by the mean and ±SD [26]. Bivariate and multivariate logistic regression was used to assess the association between the dependent variables and independent variables and the odds ratio was used to assess the strength of association. In bivariate logistic regression model *P* value less than 0.2 were considered to be confounders. Then, the confounded factors were entered and analyzed by multivariate logistic regression to measure the strength of associations with *P*<0.05 were significantly associated with the prevalence of *G*. *lamblia* infection [27].

## Results

### Microscopic examination of *G*. *lamblia* isolated from stool specimens

**Fig 2:**
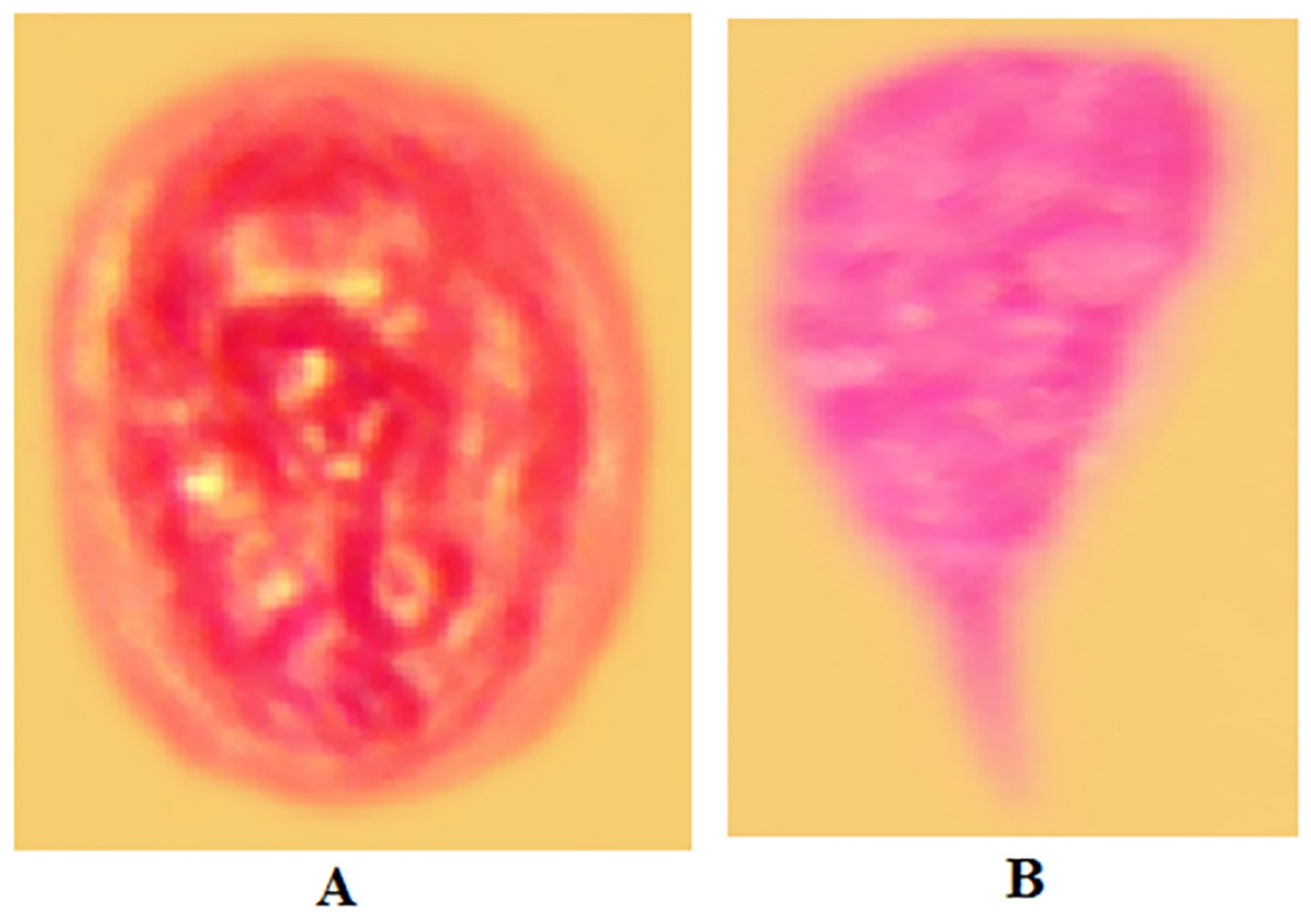
Microscopic examination of *G*. *lamblia* using wet mount and formol-ether concentration technique. A, Cyst form; B, Trophozoite form.

*Gardia lamblia* parasite isolated from fresh stool samples collected from school children were examined under microscope. Microphotographs of the specimens were captured using a digital camera. Finally, colouring of both cyst and trophozoite stages were performed using Adobe Photoshop Colour Balancing Tools (Fig 2).

### Prevalence and socio-demographic factors of the study subjects

The overall prevalence of *G*. *lamblia* infection in selected elementary schools was 27.1%. From the total of 422 expected participants about 409 were successfully collected and included into the final analysis, which gave the response rate of 97%. Thirteen 13 (3%) students were excluded from the study due to insufficient and dried stool sample delivery for the laboratory investigation. The mean age of the respondents was 10.4 (±2.8). Out of the 409 students sampled, 111 (27.1%) were infected with *G*. *lamblia* while, 298 (72.9%) were negative for infection. Of which, about 201 (49.1%) and 208 (50.9%) of the respondents were males, and females, respectively. As such, 61 (30.3%) male students and 50 (24.0%) female students were observed to be infected with *G*. *lamblia* infection in the proposed work (Table 2).

**Table 2:** Socio-demographic characteristics of government elementary school students of Lok Abaya town, Sidama zone, SNNPR, Ethiopia.

Variables	Category	Status of *G*. *lamblia* infection		Total (N)
		Negative	Positive	
Sex	Male	140 (69.7%)	61 (30.3%)	201 (49.1%)
	Female	158 (76.0%)	50 (24.0%)	208 (50.9%)
Name of school	Hantate	155 (87.6%)	22 (12.4%)	177 (43.3%)
	Chelbesa	91 (66.9%)	45 (33.1%)	136 (33.3%)
	Aregeda	52 (54.2%)	44 (45.8%)	96 (23.4%)
Age category	6–9 years	128 (70.7%)	53 (29.3%)	181 (44.3%)
	10–14 years	130 (72.2%)	50 (27.8%)	180 (44.0%)
	≥ 15 years	41 (83.81%)	07 (18.9%)	48 (11.7%)
Class category	Grade 1 –Grade 4	180 (71.7%)	71 (28.3%)	251 (61.4%)
	Grade 5 –Grade 8	118 (74.7%)	40 (25.3%)	158 (38.6%)
Family size	1–3 members	178 (76.4%)	55 (23.6%)	233 (57.0%)
	≥ 4 members	155 (37.9%)	21 (31.8%)	176 (43.0%)
Fathers educational status	Unable to read and write	82 (64.1%)	46 (35.9%)	128 (31.3%)
	Able to read and write	85 (72.0%)	33 (28.0%)	118 (28.9%)
	Elementary school	54 (78.3%)	15 (21.7%)	69 (16.9%)
	Secondary and preparatory school	26 (72.2%)	10 (27.8%)	36 (8.8%)
	Diploma and above	51 (87.9%)	7 (12.1%)	58 (14.2%)
Mothers educational status	Unable to read and write	100 (62.9%)	59 (37.1%)	159 (38.9%)
	Able to read and write	89 (74.2%)	31 (25.0%)	120 (29.3%)
	Elementary school	41 (87.2%)	6 (12.8%)	47 (11.5%)
	Secondary and preparatory school	25 (69.4%)	11 (30.6%)	36 (8.8%)
	Diploma and above	43 (91.5%)	4 (8.5%)	47 (11.5%)
Fathers Occupation	Merchant	94 (76.4%)	29 (23.6%)	123 (30.1%)
	Labor	20 (76.9%)	6 (23.1%)	26 (6.4%)
	Government worker	60 (85.7%)	10 (14.3%)	70 (17.1%)
	Farmer	124 (65.3%)	66 (34.7%)	190 (46.5%)
Mothers occupation	House wife	78 (64.5%)	43 (35.5%)	121 (29.6%)
	Merchant	87 (77.0%)	26 (23.0%)	113 (27.6%)
	Labor	52 (88.0%)	7 (11.9%)	59 (14.4%)
	Government worker	12 (70.6%)	5 (29.4%)	17 (4.2%)
	Farmer	69 (69.7%)	30 (30.3%)	99 (24.2%)

Majority of study participants, 251 (61.4%) belong to grade 1 to grade 4. Within the 3 age groups used in the study, 181 (44.3%) fall under the age of 6–9 years, 180 (44.0%) belongs to the age group of 10–14 years, and 48 (11.7%) students were ≥15 years. From those study participants, the majority 53 (29.3%) and least 7 (18.9%) students were infected with the age 6–9 and ≥15years, respectively. Among 409 students, 177, 136, and 96 were from Hantate, Chelbesa, and Aregeda elementary school, respectively. One of the schools was from an urban area (Hantate) and others two were located in rural area (Chelbesa and Aregeda). The highest prevalence was observed in two rural area schools i.e. Aregeda 44 (45.8%) and Chelbesa 45(33.1%) while, Hantate had the least prevalence rate 22 (12.4%) of infection (Table 2).

### Behavioral and environmental related risk factors related to *G*. *lamblia* infection

Many of the students 294 (71.9%) reported that their drinking water source was tap water and 234 (68.9%) students responded that their drinking water was from the raw sources which is mostly untreated. All of the study participants were informed that there is a toilet at their home. Majority of the subjects, 316 (77.3%) of students were unaware about Giardiasis infection and 406 (99.3%) participants used good sanitary conditions at their home as well as outside their home (Table 3).

**Table 3:** Behavioral and environmental related risk factors related to *G*. *lamblia* infection of government elementary school students from Lok Abaya town, Sidama zone, SNNPR, Ethiopia.

Variables	Category	Status of *G*. *lamblia* infection		Total (N)
		Negative	Positive	
Source of drinking water	River	13 (68.4%)	6 (31.6%)	19 (4.6%)
	Stream	3 (75.0%)	1 (25.0%)	4 (1.0%)
	Tap	250 (85.1%)	44 (14.9%)	294 (71.9%)
	Well	-	-	-
	Pond	19 (38.8%)	30 (61.2%)	49 (12.0%)
	River	23 (53.5%)	20 (46.5%)	43 (10.5%)
Water storage facility	Tanks	23 (57.5%)	17 (42.5%)	40 (9.8%)
	Plastic container (Jeri-cans)	203 (72.0%)	79 (28.0%)	282 (68.9%)
	Bucket	42 (95.5%)	2 (4.5%)	44 (10.8%)
	Clay pots	30 (69.8%)	13 (30.2%)	43 (10.5%)
Coverage of water container	Covered	292 (72.8%)	109 (27.2%)	401 (98.0%)
	Uncovered	6 (75.0%)	2 (25.0%)	8 (2.0%)
Water treatment at home	Yes	159 (90.9%)	16 (9.1%)	175 (42.8%)
	No	139 (59.4%)	95 (40.6%)	234 (57.2%)
Presence of toilet at home	Yes	298 (72.9%)	111 (27.1%)	409 (100.0%)
	No	-	-	-
Water availability for toilet	Yes	172 (90.0%)	19 (9.9%)	191 (46.7%)
	No	126 (57.8%)	92 (42.2%)	218 (53.3%)
Sanitary condition at home and outside	Yes	298 (73.4%)	108 (26.6%)	406 (99.3%)
	No	0 (0.0%)	3 (100.0%)	3 (0.7%)
Sewage disposal	Garbage pit	65 (95.6%)	3 (4.4%)	68 (16.6%)
	Outside the compound	162 (62.1%)	99 (37.9%)	261 (63.8%)
	Dust bin	71 (88.8%)	9 (11.3%)	80 (19.6%)
Habit of hand washing before feeding	Yes	297 (72.8%)	111 (27.2%)	408 (99.8%)
	No	1 (100.0%)	0 (0.0%)	1 (0.2%)
Habit of hand washing after feeding	Yes	273 (73.2%)	100 (26.8%)	373 (91.2%)
	No	25 (69.4%)	11 (30.6%)	36 (8.8%)
Hand washing materials used after defecation	Water only	63 (15.4%)	57 (67.9%)	120 (29.3%)
	Soap and water	187 (84.2%)	35 (15.8%)	222 (54.3%)
	Ash and water	59 (88.1%)	8 (11.9%)	67 (16.4%)
Habit of eating unwashed fruits and uncooked vegetables	Yes	146 (66.4%)	74 (33.6%)	220 (53.8%)
	No	152 (80.4%)	37 (19.6%)	189 (46.2%)
Dirt in finger nails	Yes	159 (68.2%)	79 (31.8%)	22 (5.4%)
	No	283 (73.1%)	104 (26.9%)	387 (94.6%)
Habit of trimming nails	Yes	270 (72.8%)	101 (27.2%)	371 (90.7%)
	No	28 (73.7%)	10 (26.3%)	38 (9.3%)
Awareness of Gardiasis	Yes	80 (86.0%)	13 (14.0%)	93 (22.7%)
	No	218 (69.0%)	98 (31.0%)	316 (77.3%)

### Bivariate and multivariate analysis of socio-demographic factors of *G*. *lamblia* infection

Among all the socio-demographic factors that are found to be associated with *G*. *lamblia* infection, bivariate and multivariate analysis revealed that the study participants with age category between 6 years to 9 years (*P* = 0.019) and the students from Aregeda primary school (*P* = 0.005) were found to be significantly associated with infection. The other covariant were not significantly associated with the *G*. *lamblia* infection (Table 4).

**Table 4:** Bivariate and multivariate analysis of socio-demographic factors of *G*. *lamblia* infections from government elementary school students of Lok Abaya town, Sidama zone, SNNPR, Ethiopia.

Variables	Category	Status of *G*. *lamblia* infection		COR (95% CI)	*P* value	AOR (95% CI)	*P* value
		Negative	Positive				
Sex	Male	140	61	0.436 (0.3230.589)	0.000	1.191 (0.516–2.746)	0.682
	Female	158	50	1	-	1	-
Name of school	Hantate	155	22	1	-	1	-
	Chelbesa	91	45	0.142 (0.091–0.222)	0.000	0.716 (0.211–2.433)	0.592
	Aregeda	52	44	0.495 (0.346–0.707)	0.000	**9.979 (1.98–15.392)**	0.005*
Age category	6–9 years	128	53	0.414 (0.301–0.570)	0.000	**2.305 (0.807–6.582)**	0.019*
	10–14 years	130	50	0.385 (0.278–0.533)	0.000	0.455 (0.045–4.573)	0.504
	≥ 15 years	41	07	1	-	1	-
Class category	Grade 1 –Grade 4	180	71	0.394 (0.300–0.519)	0.000	0.823 (0.252–2.692)	0.747
	Grade 5 –Grade 8	118	40	1	-	1	-
Family size	1–3 members	178	55	0.309 (0.228–0.418)	0.000	0.816 (0.287–2.324)	0.704
	≥ 4 members	155	21	1	-	1	-
Fathers educational status	Unable to read and write	82	46	0.388 (0.260–0.580)	0.000	1.353 (0.317–5.776)	0.683
	Able to read and write	85	33	0.278 (0.157–0.492)	0.000	4.022 (0.579–17.919)	0.159
	Elementary school	54	15	0.385 (0.185–0.798)	0.010	2.353 (0.221–15.088)	0.479
	Secondary and preparatory school	26	10	0.137 (0.062–0.302)	0.000	1.577 (0.069–16.015)	0.775
	Diploma and above	51	7	1	-	1	-
Mothers educational status	Unable to read and write	100	59	0.348 (0.231–0.524)	0.000	1.277 (0.257–6.341)	0.765
	Able to read and write	89	31	0.146 (0.062–0.345)	0.000	1.080 (0.102–11.394)	0.949
	Elementary school	41	6	0.440 (0.217–0.894)	0.023	4.942 (0.458–13.380)	0.188
	Secondary and preparatory school	25	11	0.093 (0.033–0.259)	0.000	1.828 (0.072–16.691)	0.715
	Diploma and above	43	4	1	-	1	-
Fathers Occupation	Merchant	94	29	1	-	1	-
	Labor	20	6	0.300 (0.120–0.747)	0.010	2.324 (0.303–17.808)	0.417
	Government worker	60	10	0.167 (0.085–0.326)	0.000	1.800 (0.128–25.225)	0.663
	Farmer	124	66	0.532 (0.295–0.717)	0.000	1.010 (0.196–5.191)	0.991
Mothers occupation	House wife	78	43	1	-	1	-
	Merchant	87	26	0.551 (0.380–0.800)	0.002	0.582 (0.133–2.540)	0.472
	Labor	52	7	0.299 (0.193–0.463)	0.000	0.753 (0.055–10.270)	0.832
	Government worker	12	5	0.135 (0.061–0.296)	0.000	0.925 (0.116–7.350)	0.941
	Farmer	69	30	0.417 (0.147–1.183)	0.100	0.265 (0.068–1.023)	0.054

*COR, crude odds ratio; AOR, adjusted odds ratio; CI, confidence interval.

* = statistically significant (*P*<0.05).

### Bivariate and multivariate analysis of behavioural and environmental factors of *G*. *lamblia* infection

Various behavioural and environmental factors were studied to see its relevance with *G*. *lamblia* infection. However, bivariate and multivariate analysis of behavioural and environmental demonstrated that study participants that consumed tap water (*P* = 0.006), washing of hands after defecation with water only (*P* = 0.040) and hand washing with soap and water after defecation (*P* = 0.000) were found to significantly correlated with infection. Apart from above factors, other studied characters were not significantly associated with *G*. *lamblia* infection (Table 5).

**Table 5:** Bivariate and multivariate analysis of behavioral and environmental factors of *G*. *lamblia* infections from government elementary school students of Lok Abaya town, Sidama zone, SNNPR, Ethiopia.

Variables	Category	Status of *G*. *lamblia* infection		COR (95% CI)	*P* value	AOR (95% CI)	*P* value
		Negative	Positive				
Source of drinking water	River	13	6	0.462 (0.175–1.214)	0.117	0.000 (0.000–0.000)	0.999
	Stream	3	1	0.333 (0.350–3.205)	0.341	0.012 (0.001–0.172)	0.001
	Tap	250	44	0.225 (0.167–0.302)	0.000	**0.011 (0.000–0.281)**	**0.006** *
	Pond	19	30	1.579 (0.889–2.805)	0.119	0.319 (0.036–2.835)	0.305
	River	23	20	1	-	1	-
Water storage facility	Tanks	23	17	0.739 (0.395–1.383)	0.345	1.294 (0.216–7.763)	0.778
	Plastic container (Jeri-cans)	203	79	0.389 (0.300–0.505)	0.000	0.248 (0.013–4.705)	0.353
	Bucket	42		0.048 (0.012–0.197)	0.000	0.748 (0.090–6.255)	0.789
	Clay pots	30	13	1	-	1	-
Coverage of water container	Covered	292	109	0.373 (0.300–0.465)	0.000	0.000 (0.000–0.000)	1.000
	Uncovered	6	2	1	-	1	-
Water treatment at home	Yes	159	16	0.683 (0.526–0.887)	0.004	2.549 (0.833–7.798)	0.101
	No	139	95	1	-	1	-
Water availability for toilet	Yes	172	19	0.110 (0.069–0.177)	0.000	2.380 (0.681–8.320)	0.174
	No	126	92	1	-	1	-
Sewage disposal	Garbage pit	65	3	0.611 (0.476–0.785)	0.000	3.911 (0.695–12.024)	0.122
	Outside the compound	162	99	0.127 (0.63–0.254)	0.000	2.064 (0.349–12.200)	0.424
	Dust bin	71	9	1	-	1	-
Hand washing materials used after defecation	Water only	63	57	2.111 (1.336–3.337)	0.001	**0.313 (0.096–1.026)**	**0.040** *
	Soap and water	187	35	0.187 (0.130–0.269)	0.000	**0.046 (0.009–0.249)**	**0.000** *
	Ash and water	59	8	1	-	1	-
Habit of eating unwashed fruits and uncooked vegetables	Yes	146	74	0.507 (0.383–0.670)	0.000	1.749 (0.652–4.690)	0.267
	No	152	37	1	-	1	-
Habit of trimming nails	Yes	270	101	0.374 (0.298–0.470)	0.000	0.739 (0.149–3.678)	0.712
	No	28	10	1	-	1	-
Awareness of Gardiasis	Yes	80	13	0.163 (0.090–0.292)	0.000	1.199 (0.361–3.989)	0.767
	No	218	98	1	-	1	-

COR, crude odds ratio; AOR, adjusted odds ratio; CI, confidence interval.

* = statistically significant (*P*<0.05).

## Discussion

Parasite infection caused by *G*. *lamblia* is one of the leading causes of death among children in the developing countries [12, 13]. Therefore, adequate information about the prevailing state is a prime requisite for epidemiological study for evaluating existing or new intervention programs. Results of our present study showed that the overall prevalence rate of *G*. *lamblia* infection among students from selected elementary schools were 27.1% (Table 2). The results are in line with the study done in Pawi special district in Benishangul-Gumuz region with a prevalence rate of 26.6% among children below 14 years old that drink water from different sources [28]. Another study that was conducted in Dilla Town elementary schools, SNNPR; Ethiopia has reported 28% rate of *G*. *lamblia* infection among school students [29]. Primary schools in western Tajikistan presented 26.4% Giardiasis infection rate having the age of 7–11 years [30]. The similarity rates of *G*. *lamblia* from the above study area might be due to socio-demographic, environmental, and behavioural conditions found around the vicinity of these regions.

However, the prevalence rate observed in this study was higher than that of the study conducted around South East of Lake Langano, Ethiopia which was around 6.2% [17]. Other reports showed that students from Dona Berber primary school located in Bahir Dar, Ethiopia has 11.4% rate of *G*. *lamblia* infection [13], in Homesha district in Benishangul-Gumuz revealed 12.65% of infection among participants [14], in Dagi primary school it was observed to be 22.8% [31], from Wukro Town, eastern Tigray, Ethiopia found 16.9% rate of infection [15], eastern region of Nepal noted 12.5% infection rate [32], study from Egypt estimated 14.5% rate of infection [33], and in Cotedivoire it was recorded as 3.9% [34]. However, the finding of our study is still higher according to the national safe environment strategy in the extension program in Ethiopia. The probable reason for this difference might be due to the variation in the socioeconomic status of study areas and could be attributed to seasonality, geographical differences, the study population size, and study design. Another possibility may be the geographic settings of the proposed areas together with crowding conditions and poor living standards of the society.

On the other hand, when comparing the result prevalence rate of *G*. *lamblia* in this study with other, it was found to be lower than other similar studies. For example, 69.4% prevalence of infection was observed among Delgi school children in North Gondar, Ethiopia [35], 33.4% rate of infection was seen in Elengaz area, Khartoum [36], 46.40% of infection was noted in Alhag Yousif area of Khartoum [37] and in Northern Iraq it was recorded as 38.5% of infection [38]. As such, these variations might be due to difference in environmental sanitation, personal hygiene, low family educational level, individual behaviour, geographical difference, the living, and the socioeconomic nature of the study subjects, diagnosis method are widely recognized risk factors accountable for the elevated prevalence of Giardiasis in most developing countries.

It was observed in our study that relatively more males were infected by *G*. *lamblia* infection 61 (30.3%) than females 50 (24.0%) (Table 2). However, no significant association was found between sex and the prevalence rate of parasite. This result was supported by a research done at South Eastern Nigeria, where the prevalence rate was higher among males 5.7% than females 4.8% [39]. In Kashmir valley from India, the infection rate was found to be higher in case of male children 10.4% than in females 6.29% [40]. Similarly, in Tirana it was reported that 55.6% of males and 44.4% of females are found to be prone to Giardiasis [41]. This variation might be due to males are more involved in playing outdoors. And also, the majority of males were moved from place to place in order to work, contaminated with soil which contains intestinal parasite protozoa during farming, playing outdoor games more frequently than females. This result was inconsistent with the study conducted at Chencha town primary school children where the prevalence rate was higher in female students 53.2% as compared to male students having the prevalence rate of 46.8% [42]. The reason for this difference might be due to the gender may or may not play a role in parasite infection depending on the region and other environmental or behavioural factors. The increased mobility of the male increases the risk of infection among them. While, females may be involved with more contact with soil and dirt during growing and harvesting vegetables and probably involved in consuming raw vegetable with prepared food more often than males.

The number of household that was greater than four members had 31.8% greater risk of having *G*. *lamblia* infection than counterparts i.e. family of 1–3 members having 23.6% chances of infection (Table 2). This is in line with the study conducted in Goiania state of Brazil [43] and in Ghana [44]. This can be associated with overcrowded conditions of household members can lead to intra-family transmission from close contacts of crowded houses [45]. It can also be due to the fact that having more children in the house could be a burden for the mother to keep her children hygiene due to shortage of time and limited resources.

The findings of our study showed that, the prevalence rate of *G*. *lamblia* infection in students with mothers and fathers unable to write and read were 59 (37.1%) and 46 (35.9%), respectively when compared to their counterparts (Table 2). But, there was no significant association observed between the parent’s educational status and the parasitic infection. In contrast, a study done in Portugal noted that the prevalence of *G*. *lamblia* infection in children with uneducated mothers was more likely to be infected with *G*. *lamblia* than those with educated mothers. Similarly, children living with fathers with no education had a 12.26 times higher risk of being infected with Giardia than those with educated fathers [46]. The reason might be due to health education can change an individual’s knowledge and educated mothers will have better knowledge on proper family planning as well as food handling, environmental, and personal hygiene, toilet use, and hand washing practices which makes educated mother eligible to handle personal hygiene among family members than that of illiterate mothers.

The prevalence of *G*. *lamblia* infection was significantly associated with some risk factors, such as rural setting of schools (AOR = 9.979, 95% CI; 1.976–15.392, *P* = 0.005), 6–9 age categories (AOR = 2.305, 95% CI; 0.807–6.582, *P* = 0.019), tap water source for drinking (AOR = 0.011, 95% CI; 0.000–0.281, *P* = 0.006), habit of washing hands after defecation with water only (AOR = 0.313, 95% CI; 0.096–1.026, *P* = 0.040) and use of soap and water (AOR = 0.046, 95% CI; 0.009–0.249, *P* = 0.000) (Table 4). The infection rates of *G*. *lamblia* were higher among the school children belonging to rural areas such as Aregeda primary school being 45.8% than that of urban areas in Hantate elementary school 12.4%. This finding is consistent with a study conducted in India which stated that, the infection rate was higher among the children belonging to rural areas (9.87%) than in children belonging to urban areas being 6.03% [40]. As the present study showed that, rural primary schools in Aregeda had 9.97 (AOR = 9.979, 95% CI; 1.976–15.392, *P* = 0.005) times more likely to be exposed with *G*. *lamblia* infection than those urban school (Table 4). The possible reason for this is the presence of relatively poor personal and environmental hygiene conditions in the Aregada primary school, poor hand washing habits among kids, unavailability and poor toilet use and unprotected water sources in the mentioned area. This finding is inconsistent with the reports from Adigrat, Ethiopia where students living in the rural area schools were less likely (AOR = 0.048, 95% CI; 0.001–1.828, *P* = 0.025) to have *G*. *lamblia* infection than those from urban regions [5].

Another study in Gurage zone, Ethiopia claimed that students attending rural school are less likely (AOR = 0.63, 95% CI; 0.42–0.96, *P* = 0.145) to have *G*. *lamblia* infection as compared to their urban area [47]. The authors suggest that the presence of relatively poor personal and environmental hygiene, poor hand washing habits, unavailability and poor toilet use and unprotected water sources in rural areas than urban as possible reasons. As the current research revealed that, prevalence rate of *G*. *lamblia* infection was slightly higher among 6–9 age groups being 53 (29.3%) and less prevalent among ≥15 age groups which was 7 (18.9%). The reason for decline in infection with increase in age may be possibly due to strengthening of the immune system and children becoming more conscious of hygienic habits. Similar studies done by Kidane et al. [15] in Wuokero claimed that the higher and least prevalence of *G*. *lamblia* infection was seen in the age groups of 6–9 and 15–18 years, respectively. But, the age category of 6–9 was significantly associated with the parasite had 2.305 (AOR = 2.305, 95% CI; 0.807–6.582, *P* = 0.019) times more likely to be exposed *G*. *lamblia* infection than counterparts (Table 4). This is in consistent with the study from India (*P* = 0.01) [40] and in Tirana [41]. Another study reported in Brazil stated that the age group of 5–10 had 1.18 (AOR = 1.18, 90% CI; 1.0–1.36; *P* = 0.002) times more likely infected with *G*. *lamblia* parasite [42] and in Malaysia it was found that students whose age was under 12 years were 5.54 (AOR = 5.54, 95% CI; 3.7–8.2, *P* = 0.00) times more likely to be infected with the parasite [48]. This finding is in accordance with previous studies reported that the highest risk was seen in young children with a decreasing risk in older children and adults. The reasons for the agreement of studies might be due to younger children are more exposed since they usually play in the open fields and follow poor personal hygiene practices.

The current study reported that, students who used tap water as a source of drinking water were 44 (14.9%) infected with *G*. *lamblia* parasite as compared to those who consume other water source (Table 5). But it is significantly associated with (AOR = 0.011, 95% CI; 0.000–0.281, *P* = 0.006). In contrast, reports from Malaysia evidenced that participants who used tap water for drinking had 3.655 (AOR = 3.655, 95% CI; 3.52–15.80, *P* = 0.050) times more likely to be exposed with *G*. *lamblia* infection [48]. The probable reason may for the contamination of tap water could be attributed to the usage of containers and utensils that might have been previously harboured with protozoan cysts during handling and storage of drinking water. Furthermore, who used tanks or other uncovered containers for drinking water collection can contribute to infection more when compared to those who preferred cleaned and covered tanks.

It was reported that children who use water only for washing their hands after defecation were 0.313 (AOR = 0.313, 95% CI; 0.096–1.026, *P* = 0.040) times less likely associated with *G*. *lamblia* infection than those who did not wash their hands after defecation (Table 5). And the use of soap and water after defecation were 0.046 (AOR = 0.046, 95% CI; 0.009–0.249, *P* = 0.000) times less likely to be infected with *G*. *lamblia* infection than students who use water only (Table 5). In contrast, another study reported in Aksum, Ethiopia finds that, school children who did not wash their hands with soap after defecation (AOR = 11.24, 95% CI; 6.73–18.78, *P* = 0.000) were eleven times more likely to acquire *G*. *lamblia* infection than children who wash their hands with soap after defecation [49]. As we observed that in the selected schools there was no water around the toilet. These conditions might give rise to increase the infection rate of *G*. *lamblia*. From the expected determinant factors of *G*. *lamblia* infection: rural setting school, age, drinking of tap water source, the habit of hand washing with water only and soap and water were significantly associated with the prevalence of the parasite (*P*< 0.05).

## Conclusion

In the current study, a total of 111 (27.1%) students were infected with *G*. *lamblia* infection and it is a common health problem among the study area. Significant predictors of *G*. *lamblia* infection were the rural school children, 6–9 age group students, tap water source for drinking, hand washing habit after defecation with water only and soap and water having *P*< 0.05 and might be related with the positive result of *G*. *lamblia* infection. The study participants that have no awareness about *G*. *lamblia* infection transmission and prevention method were highly infected than those who are having knowledge about the parasite but there was no statistically significant association observed between awareness about *G*. *lamblia* infection and prevalence of *G*. *lamblia* infection in this study. Thus, this study is the first approach to determine the prevalence of *G*. *lamblia* infection and its associated factors from selected area. As such, improvement of general standards of sanitation through the installation of suitable sewage treatment, disposal facilities and provision of tap water supply as pre-requisites for successful prevention and control are necessary. Establishment and maintenance of a network for treatment of *G*. *lamblia* infection, the provision of health education program in primary schools and health initiatives or clubs in school should be introduced and should focus on primary diseases prevention. Awareness should be created on *G*. *lamblia* transmission and treatment for students and their parents. Local health sector and any concerned bodies should collaborate with school health programs for delivering health education to increase the knowledge, attitude, and practice of school children to minimize or prevent the transmission of this parasite infection and may possibly eradicate this predicament.

## Supporting information

S1 File Questionnaire used in the proposed research work. (PDF)
