# Supplementary material for: Assessment of prevalence of Giardia lamblia infection and its associated factors among government elementary school children from Sidama zone, SNNPR, Ethiopia
Source: PLoS One. 2022 Mar 15;17(3):e0264812. doi: 10.1371/journal.pone.0264812 (PMC8923448; doi:10.1371/journal.pone.0264812)
Supplement: S1 File — (PDF) [file pone.0264812.s001.pdf]

DILLA UNIVERSITY

SCHOOL OF GRADUATE STUDIES

COLLEGE OF NATURAL AND COMPUTATIONAL SCIENCES

DEPARTMENT OF BIOLOGY

Appendix I: Questionnaire (English version)

Introduction: This questionnaire is prepared to assess the prevalence rate and associated risk factors of *Giardia lamblia*infections among government elementary school children in Loka Abaya Woreda, Sidama zone, SNNPR, Ethiopia, 2019. So, after you read each of the following questions carefully please answer by circle the numbers. It is your full right to participate, withdraw or refuse to be involved in this study, but your real honest participation is very important for introducing intervention programs, controlling, preventing and elimination of the disease progression.

Thank you in advance for your cooperation!

Part I: Socio demographic factors

1. Sex: 1. Male

2. Female

2. Age: 1. 6-9

2. 10-14

3. ≥15

3. Name of school: 1. Hantante 2. Chelbesa 3. Aregeda

4. Class: _________________

5. Father's educational status

1. Unable to write and read

2. Able to write and read only

3. Elementary school

4. Secondary and preparatory school

5. Diploma and above

6. Mother’s educational status

1. Unable to write and read

2. Able to write and read only

3. Elementary school

4. Secondary and preparatory school

5. Diploma and above

7. Father’s Occupation

1. Merchant 3. Government worker

2. Laborer4. Farmer 5. Other specify------

8. Mother’s occupation

1. House wife

2. Merchant

3. Government worker

4. Daily laborer

5. Farmer

6. Others ________________

9. Number of household members (Family size)?______

Part II: Environmental factors

10. Where is your main source of water?

1. River water
2. Stream water
3. Tap water
4. Well water
5. Pond water
6. Rain water
7. Others specify--------

11. How water at home is stored?

1. Tanks
2. Jericans
3. Bucket
4. Clay pots
5. Others specify-----

12. Is this water storage container usually covered?

1. Yes
2. No

13. Do you treat drinking water at home?

1. Yes
2. No

14. Is there a latrine at your home?

1. Yes
2. No

15. If yes, is their availability of water in the latrine?

1. Yes
2. No

16. Did you use good sanitary conditions in your home and environment?

1. Yes
2. No

17. Where do you dispose off house hold waste?

1. Garbage pit
2. Outside the compound
3. Dust bin
4. Others specify-----------

Part III: Behavioral factors

18. Do you wash your hand before eating food?

1. Yes

2. No

19. Do you wash your hand after defecation?

1. Yes

2. No

20. If yes, what do you use for washing?

1. Water only

2. Soap and water

3. Other -------------

21. Do you eat raw vegetables and fruit without washing and cooking?

1. Yes

2. No

22.Is there any dirty particles in your fingers nails?

1. Yes

2. No

23. Do you have a habit of trimming nail?

1. Yes

2. No

24. Do you have awareness about giardiasis?

1. Yes

2. No

25. Status of *Giardia lamblia*? 1. Negative

2. Positive

ዲላዩኒቨርሲቲ

የድህረ-ምረቃት/ቤት

የተፈጥሮእናቀመረሳይንስኮሌጅ

ሥነ-ህይወትትምህርትክፍል (ጀነራልባዮሎጂ)

መመሪያ፡ይህቃለመጠይቅየተዘጋጀዉየጃርዲያየሥርጭትመጠንእናየበሽታዉንተያያዥመንስኤዎችበሎካአ ባያወረዳበሚገኙየመንግስትአንደኛደረጃት/ቤቶችለማጥናትነዉ፡፡በመሆኑምእርስዎከዚህበታችየተዘረዘሩትን ቃለመጠይቆችበጥንቃቄካነበቡበኋላመልስዎንበማክበብይመልሱ፡፡

ቃለመጠየቁንስለሞሉእናመሰግናለን!

Appendix II: Questionnaire (Amharic Version)

ክፍል I: ስለተማሪዎችማህበራዊሁኔታ

ጾታ :- 1. ወንድ 2.ሴት

እድሜ :- 1. 6- 9 2. 10-14

3. 15- 18 4. ከ 18 አመትበላይ

ክፍል____________

1. የወላጅአባትየትምህርትደረጃ

1. ማንበብናመጻፍአይችሉም

2. ማንበብናመጻፍብቻ

3. አንደኛደረጃ

4. ሁለተኛደረጃናመሰናዶ

5. ከዲፕሎማበላይ

2. የወላጅእናትየትምህርትደረጃ

1. ማንበብናመጻፍአይችሉም

2. ማንበብናመጻፍብቻ

3. አንደኛደረጃ

4. ሁለተኛደረጃናመሰናዶ

5. ከዲፕሎማበላይ

3. የወላጅአባትስራ

1. ነጋዴ

2. የመንግስትሰራተኛ

3. የቀንሰራተኛ

4. አርሶአደር

5. ሌላካለ_________

4. የወላጅእናትስራ

1. የቤትእመቤት

2. ነጋዴ

3. የመንግስትሰራተኛ

4. የቀንሰራተኛ

5. አርሶአደር

6. ሌላካለ__________

5. የቤተሰብብዛት --------------------

ክፍል II: ስለአካባቢያዊሁኔታዎች

6. ዉሃከየትያገኛሉ?

1. ከወንዝ

2. ከምንጭ

3. ከቧንቧ

4. ከጉድጓድ

5.ከኩሬ

6. ሌላካለ________

7. የምትጠቀሙትዉሃየተከደነነዉ?

1. አዎ

2. አይደለም

8. በቤታችሁዉሃበምንይጠራቀማል?

1. በጋን

2. ጀሪካን

3. በርሜል

4. ሌላካለ_________

9. የዉሃማጠራቀሚያእቃወቹክዳንአለዉ

1. አዎ

2. የለም

10. የምትጠጡትንዉሀታክማላችሁ?

1. አዎ

2. የለም

11. በቤትዎዉስጥሽንትቤትአለ ?

1. አዎ

2. የለም

12. ጥያቄቁጥር ‘10’ ላይመልስዎአዎከሆነ፤በሽንትቤትዎዉሀአለ?

1. አዎ

2. የለም

13. ከቤታችሁቆሻሻንእንዴትታስወግዳላችሁ?

1. በበርሜል

2. ከግቢዉጭእንዲወጣበማድረግ

3.በጉድጋድ

4. ሌላካለ______

ክፍል III: ስለተሳታፊዎችባህሪበተመለከተ

14. ምግብከመብላትዎበፊትእጅዎንይታጠባሉ?

1. አዎ

2. የለም

15. ሽንትቤትከተጠቀሙበኋላእጅዎንይታጠባሉ ?

1. የለም 2. በዉሀብቻ 3.በዉሃናበሳሙና4.በአመድናበዉሀ

16. ያልታጠበአትክልትናፍራፍሬይመገባሉ?

1. አዎ

2. የለም

17. በጥፍርዎዉስጥቆሻሻአለ ?

1. አዎ

2. የለም

18. ጥፍርዎንየመቁረጥልምድአለዎት?

1. አዎ

2. የለም

19. ስለጃርዲያግንዛቤአለዎት?

1. አዎ

2. የለም

DILLU YUNIVERISITE

LAYINKI DIGIRE ROSIMINE

KALAQAMUNNA SHALLAGOTE SAYIINSE KOLLEEJE

LUBBO AFFIDHINO KALAFAMA ROSU HANDAARA

QAAGISHA: - tini xa'mo qixaalsinohu jardiyu xiwani taraawote bikkana hattono xiueanle koni kaima lokka abbaayyu woradi giddo afantano mangistete umi dirimi rosiminna giddo xiinxallo assateeti koni daafira koni worooni titirote shiqino xa'mo wodanini nababihu gedensaani xa'mote albaani no shoolu midaadi qoqouli gido koni malaatini dawaro qoli.

Xa'mo tene wonshoto/ta daafo

Galanteemo!

Appendix III: Questionnaire (Sedamic Version)

XA'MO:-

GAFA I: - rosaanote dagoemitete gara/mahiberawe huneeta/

Kooltee: 1. labano 2. meyaate

Diro 1.6-9 2.10-14 3.15-18 4. 18 alehigino

Kifile----------------------------

1. Illo anisilanise rosu dara

    1. Nabbabana borrassa didandaano
    2. Nabbabana borrassa calle dandaano
    3. Umi dirime
    4. 2 ki dirima
    5. Digilomu ale

2. Amate rosu deera

    1. Nabbabana borreessa didandiitanno

2. Nabbabana borreessa calla dandiitano
3. Umi dirima
4. 2^{ki} dirima
5. Dipl’loomu ale

3. Annu loessi: 1. Daddalo 2. Mangistete loosso

3. Barru loesso 4.baatto loossire galino

5. Woleno--------

4. Amate loossi: 1. Loossu dinose 2. Daddalo

3. Managistete loosso 4. Barru loosso

5. Baatte loossidhe galitino 6. woleno ----------------

GAFA. II: qooxxessu gara:

5. Waa mamini afidhinanni?

1. Laguni 2. Daadda buichoni

3. Banbuni 4. Baleteni /Ittisuni/

5. wolene--------------------

6. Horonsidhinanni wayi garuni tu’nooniho? 1. Ee 2. Dee’ni

7. Minine giddo waa mayi uduunichira wodhinanni?

1. Googidete 2. Jarikaanuni

3. Barmeelete 4. Wole--------

8. Waa wodhitinanni udunichi tuana afirino?

1. Ee afirino 2. Diafirino

9. Aginanni waa akammani? 1. Ee akkamani 2. Diakkamani

10. Shumate mini noon’ee? 1. Ee no 2. Dino

11. Alleeni xa’mo xiiro (10) aana dawaroki ‘Ee no ‘ ikkituro, shumate mine wayi heerano?

1. Ee herano 2. Dino

12. Minini fine fudhini ishine mayi garini huntinanni?

1. Barmeelete 2. Hoowete gobaani huneemo 3. Wole---------

GAFA III: - Xa’mote beeqaano akkata lainohuni

13. Sagale itfkira albaani angal hayishirato/ta? 1. Ee 2. Dihayishereemo/ma

14. Shumate mine horonsidne Kaitori / tara anga hayishirato / rata?

1. Dihayishireemo/ma 2. Wayini calla

3. Wayininna samuninni hayishireemo/ma

15. Hayishantinoki akakilitena firafire ittato Ha?

1. Ee ittemo/ma 2. Diitteemo/ma

16. Culunqaki giddo xuru no? 1. Ee no 2. Dino

17. Culunqa mudhate resichinoohe? 1. Ee noe 2. Dinoe

18. Jardiyu Xiwani daafo afootori / tari no?

1. Ee affoomo/ ma 2. Dino

Appendix IV: Consent Form

I--- (Name of parent or legal guardian)

Having full capacity to consent for---------------------------------- (Name of the pupil) who is under 18 years, do hereby volunteer to participate in the research study entitled ‘Prevalence and associated risk factors of*Giardia lamblia* infection among school children at Loka Abaya Woreda,Sidama Zone, SNNPR, Ethiopia’. I understand that my child will be;

- Asked questions related to knowledge and practice of personal hygiene and sanitation.
- Required to provide stool specimen for investigation of *Giardia lamblia*.

I have also been informed that;

- Participation is entirely voluntary and the pupil can withdraw at will at any time.
- The findings will not be given to any unauthorized person.
- There will be no financial gain or loss to me for participating in the study.

Name-- (Pupil’s parent or legal guardian)

Signature---

Date---
